# Evaluation of the effectiveness of superficial parotidectomy and partial superficial parotidectomy for benign parotid tumours: a meta-analysis

**DOI:** 10.1186/s40463-023-00679-w

**Published:** 2023-12-22

**Authors:** Hai-Tao Liu, Wei-Peng Jiang, Gang Xia, Jia-Min Liao

**Affiliations:** Department of Oral and Maxillofacial Surgery, First People’s Hospital of Jiujiang City, No.48 of Taling South Street, Jiujiang, 332000 Jiangxi China

**Keywords:** Benign parotid tumour, Superficial parotidectomy, Partial superficial parotidectomy, Meta-analysis

## Abstract

**Objective:**

To quantify the results of superficial parotidectomy (SP) and partial SP (PSP) for benign parotid tumours using a systematic evaluation method.

**Methods:**

A systematic search of English and Chinese databases (PubMed, Web of Science, Cochrane Library, China Knowledge Network, Wanfang and Vipshop) was conducted to include studies comparing the treatment outcomes of SP with PSP.

**Results:**

Twenty-three qualified, high-quality studies involving 2844 patients were included in this study. The results of this study showed that compared to the SP surgical approach, the PSP surgical approach reduced the occurrence of temporary facial palsy (OR = 0.33; 95% confidence interval [CI] 0.26–0.41), permanent facial palsy (OR = 0.28; 95% CI 0.16–0.52) and Frey syndrome (OR = 0.36; 95% CI 0.23–0.56) in patients after surgery, and the surgery operative time was reduced by approximately 27.35 min (95% CI − 39.66, − 15.04). However, the effects of PSP versus SP on salivary fistula (OR = 0.70; 95% CI 0.40–1.24), sialocele (OR = 1.48; 95% CI 0.78–2.83), haematoma (OR = 0.34; 95% CI 0.11–1.01) and tumour recurrence rate (OR = 1.41; 95% CI 0.48–4.20) were not statistically significant.

**Conclusion:**

Compared with SP, PSP has a lower postoperative complication rate and significantly shorter operative time, suggesting that it could be used as an alternative to SP in the treatment of benign parotid tumours with the right indications.

**Graphical abstract:**

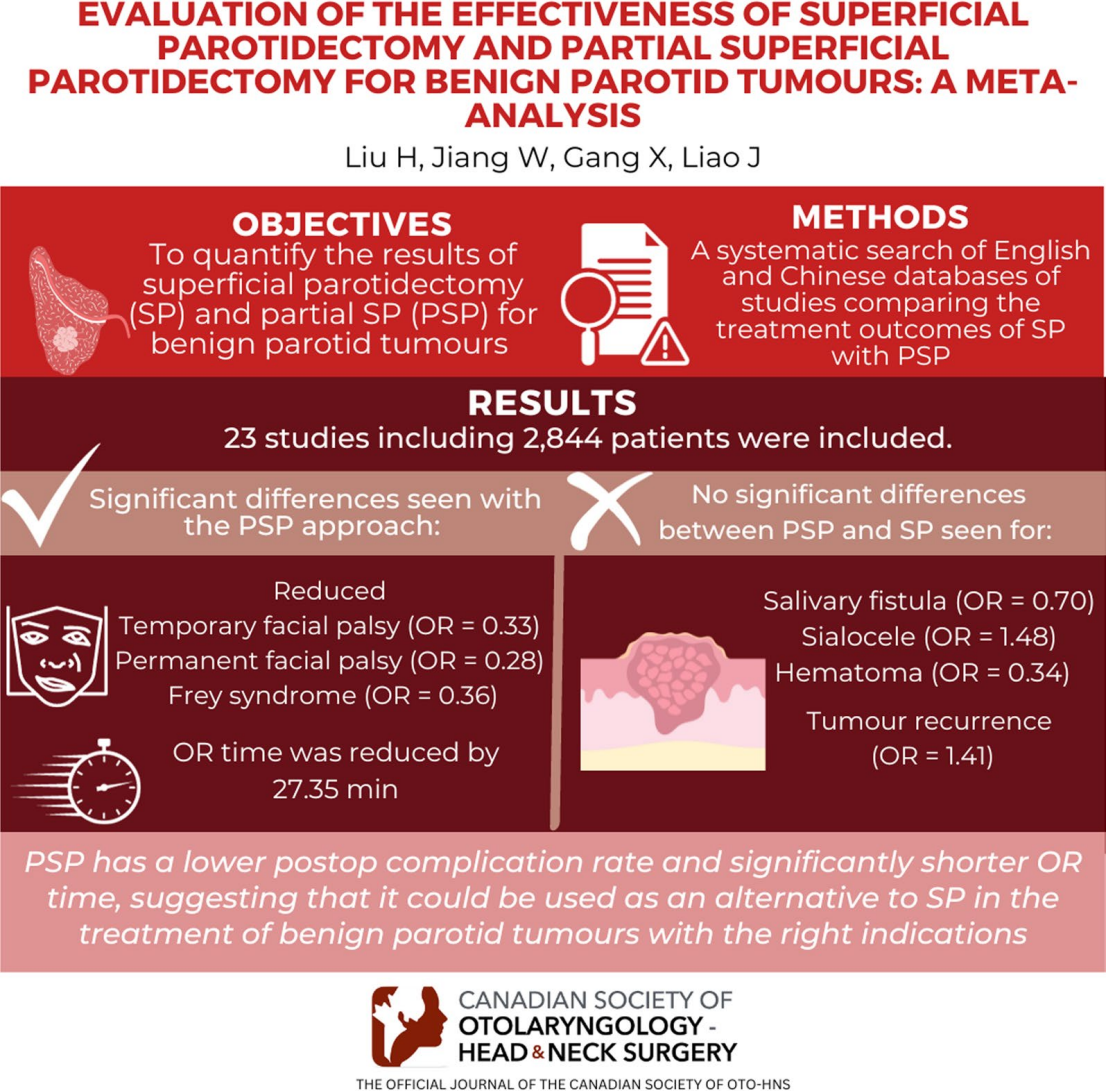

## Introduction

Data from previous studies show that the most common and serious disease of salivary gland tissue is salivary gland tumours, which account for approximately 3% of all head tumours [1], with benign parotid tumours being the most prevalent type of salivary gland tumour at an incidence of 75–80% [2, 3]. The treatment of benign parotid tumours remains a critical clinical problem.

Surgical resection therapy is the mainstay of treatment of benign parotid tumours. In the 1950s, Martin et al. [4] pioneered the utilization of superficial parotidectomy (SP) using the facial nerve as an anatomical landmark. This surgical approach gradually became the basic surgical procedure for the treatment of benign parotid tumours [5]. However, although this procedure has reduced the tumour recurrence rate to 2%, it is still a drawback for facial nerve palsy and the impact on the patient’s appearance [6]. With the advancement of medical technology and the efforts of both doctors and patients to preserve the function and appearance of the organ, partial SP (PSP) has been proposed and practiced by scholars. Partial SP differs from SP in that the resection area is smaller and it can better protect the secretory function of the gland and maintain facial aesthetics [7, 8]. However, there are still reported outcomes of tumour recurrence, Frey syndrome and facial nerve palsy [9–11]. Although surgeons generally agree that the risk of complications after parotidectomy is related to the extent of the parotidectomy, sufficient evidence is still lacking. Therefore, the aim of this study is to understand the effect of PSP and SP on postoperative complications in patients using meta-analysis and to provide a selection of surgical options for benign parotid tumours.

## Method and materials

### Search strategy

Following the PRISMA 2020 statement [12], three English databases, including PubMed, Web of Science and Cochrane Library, and three Chinese databases, including China Knowledge Network, Wanfang and Vipshop, were systematically searched. The search time was from the establishment of the database to 10 April 2023. The English database search strategy included the following keywords: ‘parotid benign tumours’ AND ‘partial parotidectomy OR limited resection of parotid gland OR regional resection of parotid gland OR superficial parotidectomy’ AND ‘partial superficial parotidectomy’. The same search terms were used for the Chinese database. In addition, the target literature was obtained by reviewing the references of the included studies.

### Inclusion and exclusion criteria

The inclusion criteria were as follows: (1) studies published in peer-reviewed journals in English and Chinese; (2) study subjects with a substantial, epithelial tumour of primary origin in the parotid gland that was benign; (3) partial superficial parotidectomy or SP performed on study subjects; (4) study outcomes of interest included at least one instance of facial palsy, recurrent outcome, Frey syndrome or salivary fistula; and (5) study was a case control or prospective study. In addition, PSP was defined as excision of the parotid tumour and 0.5–1.0 cm of normal gland surrounding the tumour without dissecting the facial nerve or dissecting only part of the facial nerve branches involved in the tumour; SP was defined as dissection of the facial nerve and excision of all or most of the superficial lobe of the parotid gland, including the tumour [13].

The exclusion criteria were as follows: (1) non-population studies; (2) conference articles, case reports and systematic reviews; (3) inadequate information on outcomes and inability to perform data analysis; (4) duplicate reports of studies in the literature; and (5) studies for which complete articles were not available.

### Literature screening and data extraction

Literature screening based on inclusion and exclusion criteria was conducted by two researchers individually, first by reading the titles and abstracts of the literature for initial screening and then by reading the full text of studies that might meet the inclusion criteria. When a disagreement occurred between the two researchers, a third researcher was consulted to reach a unified opinion. After the literature screening was completed, two researchers performed the data extraction based on a standard data extraction form. The extracted information included literature information, the demographic characteristics of the study population, the mode of surgery, the study duration and the outcome events.

### Quality evaluation

The Newcastle–Ottawa Scale (NOS) [14] was used to evaluate the quality of the literature based on eight items including the representativeness of the study population, comparability between groups, adequacy of the study’s evaluation of outcomes, adequacy of the follow-up time and completeness of the follow-up, with a high score of nine. A total score of seven and above was for high-quality literature, and a score of five and below was for low-quality articles.

### Statistical analysis

The statistical analysis was performed using the Revman 5.3 software. Effect sizes were expressed as ratio ratios (ORs) for count data and mean differences (MDs) for measures, and 95% confidence intervals (CIs) were used to estimate the interval range of effect sizes. Heterogeneity tests were performed using *I*^*2*^ statistics and the Q test to determine the magnitude of heterogeneity. With *I*^2^ < 50% or *P* > 0.1, the included literature was considered homogeneous and was analysed using a fixed effects model (Mantel–Haenszel); if *I*^2^ > 50% or *P* ≤ 0.1, the included studies were considered to have poor homogeneity, and a random effects model (DerSimonian–Laird) was used for analysis. If heterogeneity was high, a sensitivity analysis was used to explore the sources of heterogeneity. A value of *P* ≤ 0.05 was considered a statistically significant difference.

## Results

### Basic characteristics of the included studies

**Fig. 1:**
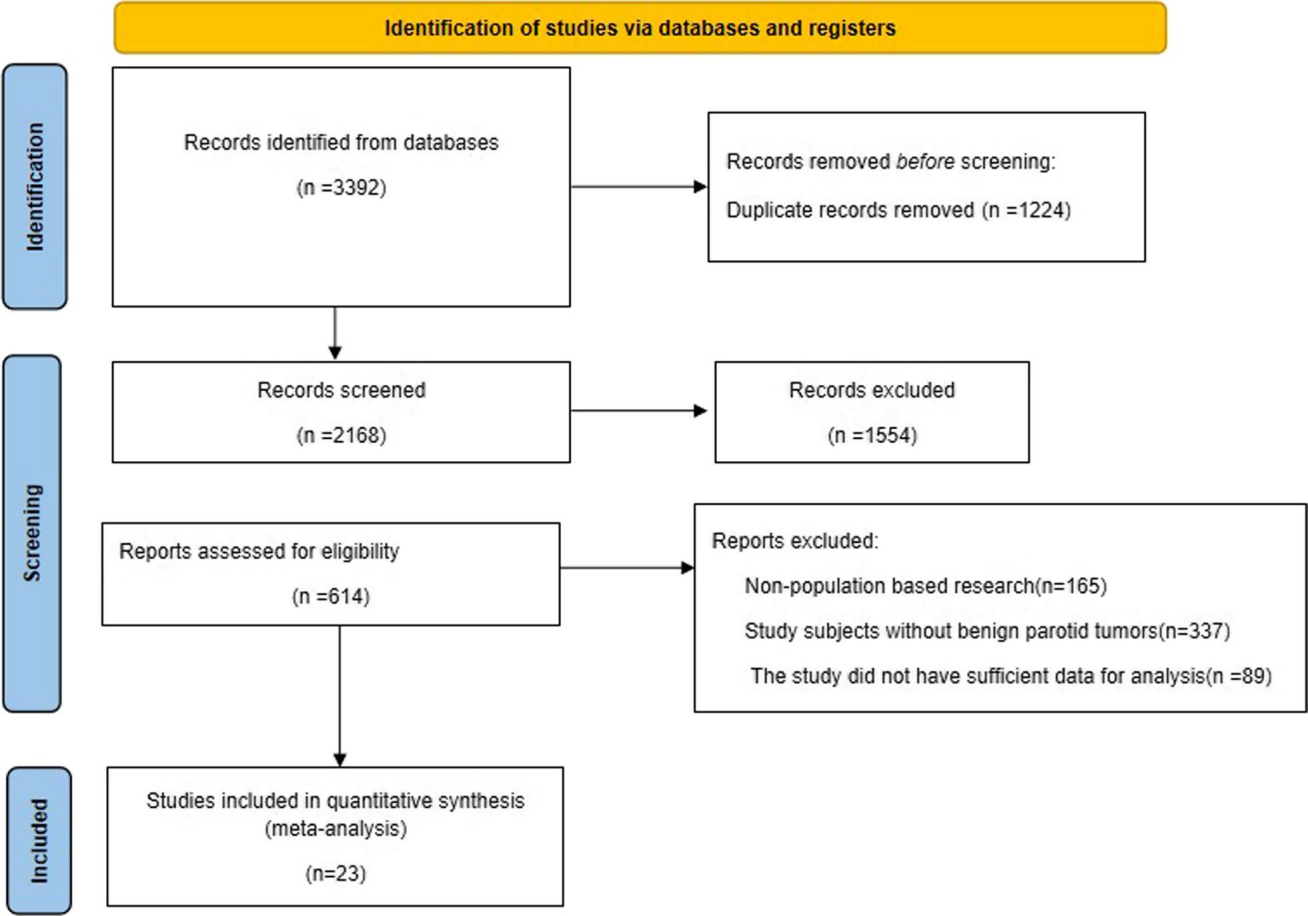
Flow chart of literature screening

After a systematic search of the English and Chinese databases, 614 publications were included in the full-text review, and in the end, a total of 23 [9–11, 15–34] publications met the inclusion criteria of this study. The literature screening process is shown in Fig. 1. All studies were published during 2002–2021; 7 studies were from China, and 18 studies were retrospective. Moreover, the 23 studies involved 2844 patients in total, of whom 1430 patients were treated with PSP and 1414 patients were treated with SP. In addition, the results of the quality evaluation of the literature showed that most of the studies had a low risk of bias with a mean NOS score of 7.04 (median: 7); more information on the basic characteristics of the literature is shown in Table 1.

**Table 1:** Basic characteristics of included studies and results of literature quality

Study	Location	Study design	Follow time	Sample (PSP/SP)	Age	Male (%)	Tumor size	Outcome	NOS
Lu, 2017	China	Retrospective study	6 months–5 years	92/76	20–74	44.05	0.8–4.0 cm	Surgery time, Tumor recurrence, Temporary facial nerve paralysis, Salivary fistula, Frey's syndrome	8
Ogreden, 2016	Turkey	Retrospective study	5 years	32/18	44.5	50		Frey's syndrome, Tumor recurrence	7
Al-Aroomi, 2021	China	Prospective study	12 months	20/35	18–77	56.4	2.8 cm	Surgery time, Temporary facial nerve, Permanent facial nerve, Frey's syndrome, Sialocele, Hematoma	7
Ruohoalho, 2017	Finland	Prospective study	12 months	53/32	20–86	44.7	0.3–5.5 cm	Temporary facial nerve, Permanent facial nerve, Frey's syndrome, Salivary fistula	8
Zhang,2013	China	Retrospective study	18 months	163/105	51	56	< = 2 cm	Tumor recurrence, Temporary facial nerve, Permanent facial nerve, Salivary flow	6
Koch, 2010	German	Retrospective study	76.7 months	34/134	50.7	54.5		Tumor recurrence, Temporary facial nerve, Permanent facial nerve, Salivary fistula	7
Li,2014	China	Retrospective study	62 months	58/71	40.8	44	< 2 cm, 2–4 cm, > 4 cm	Tumor recurrence, Temporary facial nerve, Permanent facial nerve, Salivary fistula, Frey's syndrome, Operative time	7
Ciuman, 2012	German	Retrospective study	> = 1 year	95/52		52		Temporary facial nerve, Permanent facial nerve, Frey's syndrome	6
Stathopoulos, 2018	Ireland	Prospective study	5 years	135/43	55.9	45	3.38 cm	Tumor recurrence, Temporary facial nerve, Permanent facial nerve, Frey's syndrome, Sialocele, Hematoma	6
Roh, 2007	Korea	Prospective study	48 months	52/49	11–82	47		Tumor recurrence, Temporary facial nerve, Permanent facial nerve, Frey's syndrome, Sialocele, Surgery time	7
Mlees, 2020	Egypt	Retrospective study	7 years	44/40	19–76	36	2.6/2.4	Tumor recurrence, Temporary facial nerve, Permanent facial nerve, Frey's syndrome, Sialocele, Hematoma, Surgery time	8
Huang, 2015	China	Retrospective study		79/241	19–83	41		Tumor recurrence, Temporary facial nerve, Permanent facial nerve, Frey's syndrome	8
Kilavuz, 2018	Turkey	Retrospective study	79 months	131/190	18–87	57		Tumor recurrence, Temporary facial nerve, Permanent facial nerve, Frey's syndrome, Salivary fistula, Hematoma, Surgery time	8
Emodi, 2010	Israel	Retrospective study	57 months	30/18	43	39.6		Tumor recurrence, Temporary facial nerve, Permanent facial nerve, Frey's syndrome, Surgery time	7
Papadogeorgakis, 2004	Greece	Retrospective study	55 months	42/17	30–77	61		Tumor recurrence, Temporary facial nerve, Permanent facial nerve, Frey's syndrome, Sialocele	7
Witt, 2009	USA	Retrospective study	1 month	100/20	57.3	45	2.1 cm	Sialocele	5
Plaza, 2015	Spain	Retrospective study	4 years	25/25	42	60	1.2–3.3 cm	Tumor recurrence, Temporary facial nerve, Permanent facial nerve, Frey's syndrome, Surgery time	6
Witt, 2002	USA	Retrospective study	8 years	20/20	42.2/45.7	43	1.94/2.0 cm	Tumor recurrence, Temporary facial nerve, Permanent facial nerve, Frey's syndrome	6
Schapher, 2021	German	Retrospective study	13.1 years	6/30	47.6	36	2.4 cm	Frey's syndrome	8
Zheng, 2019	China	Retrospective study	3 years	91/92	49/48	56	2.6/2.7 cm	Tumor recurrence, Temporary facial nerve, Permanent facial nerve, Sialocele, Frey's syndrome, Surgery time	7
Wong, 2018	New Zealand	Prospective study	31.58 weeks	56/40	18–86	46.5		Temporary facial nerve, Permanent facial nerve, Salivary fistula, Sialocele, Frey's Syndrome, Hematoma	8
Gao, 2017	China	Retrospective study	29.8 months	50/49	18–85	57.6	2.96/2.78	Temporary facial nerve, Permanent facial nerve, Frey's syndrome, Salivary fistula, Surgery time	7
Eski, 2018	Turkey	Retrospective study	41.79 months	22/17	51/57	51.3	2.5 cm	Tumor recurrence, Temporary facial nerve	8

Age is expressed as the mean or range (minimum–maximum)

### Facial nerve palsy

**Fig. 2:**
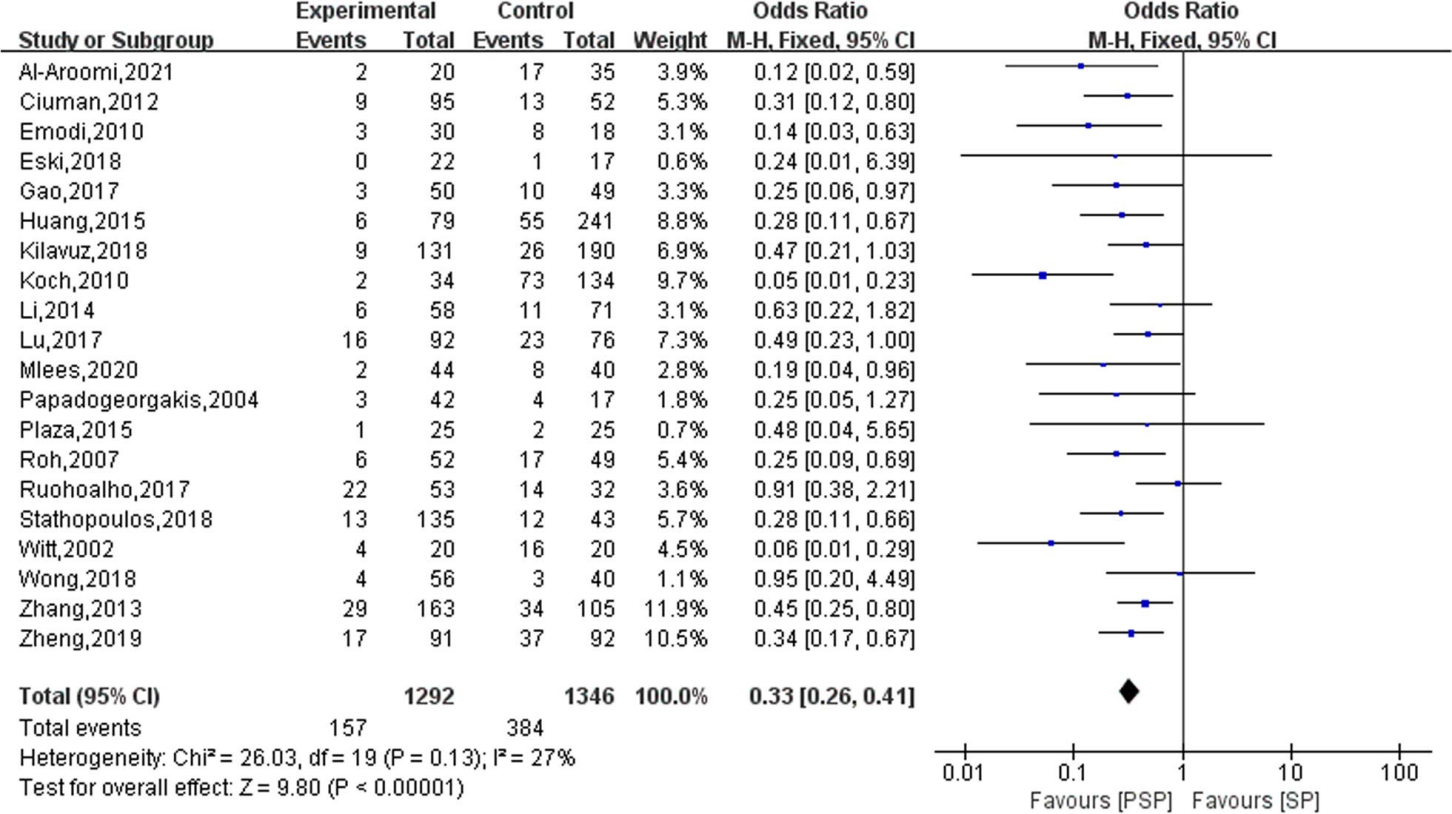
The effect of PSP and SP on patients with transient facial palsy

Twenty studies reported on the outcome of transient facial palsy occurring in the study subjects after surgery, in 1,292 patients who were treated with PSP and in 1,346 patients who were treated with SP. The results of the heterogeneity evaluation showed good homogeneity between the included studies (*I*^2^ = 27%, *P* = 0.13), and the combined effect size was calculated using a fixed effects model. The meta-analysis results showed that patients had a lower risk of developing transient facial palsy after treatment with PSP compared to SP treatment modalities (OR = 0.33; 95% CI 0.26–0.41), as shown in Fig. 2.

**Fig. 3:**
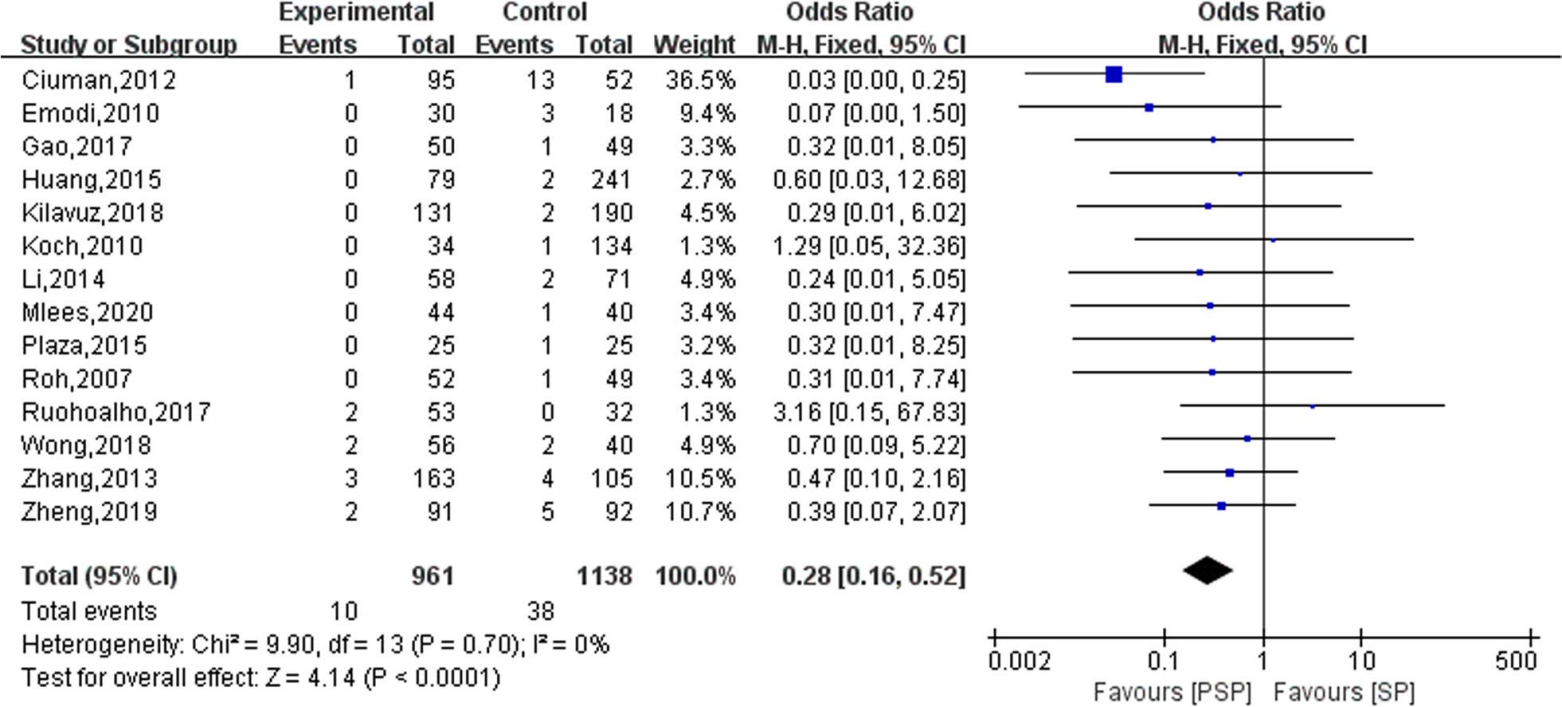
The effect of PSP and SP on permanent facial palsy in patients

Eighteen studies reported on the outcomes of permanent facial palsy occurring in the study subjects after surgery, of which four studies did not identify any patients who developed permanent facial palsy during the follow-up [11, 20, 26, 29]. Thus, only the remaining 14 studies were meta-analysed. The results of the heterogeneity evaluation showed no heterogeneity between the included studies (*I*^2^ = 0%, *P* = 0.70), and the combined effect size was calculated using a fixed effects model. The results of the meta-analysis showed that the PSP treatment modality reduced the occurrence of permanent facial palsy in patients after surgery (OR = 0.28; 95% CI 0.16–0.52), as shown in Fig. 3.

### Frey syndrome

**Fig. 4:**
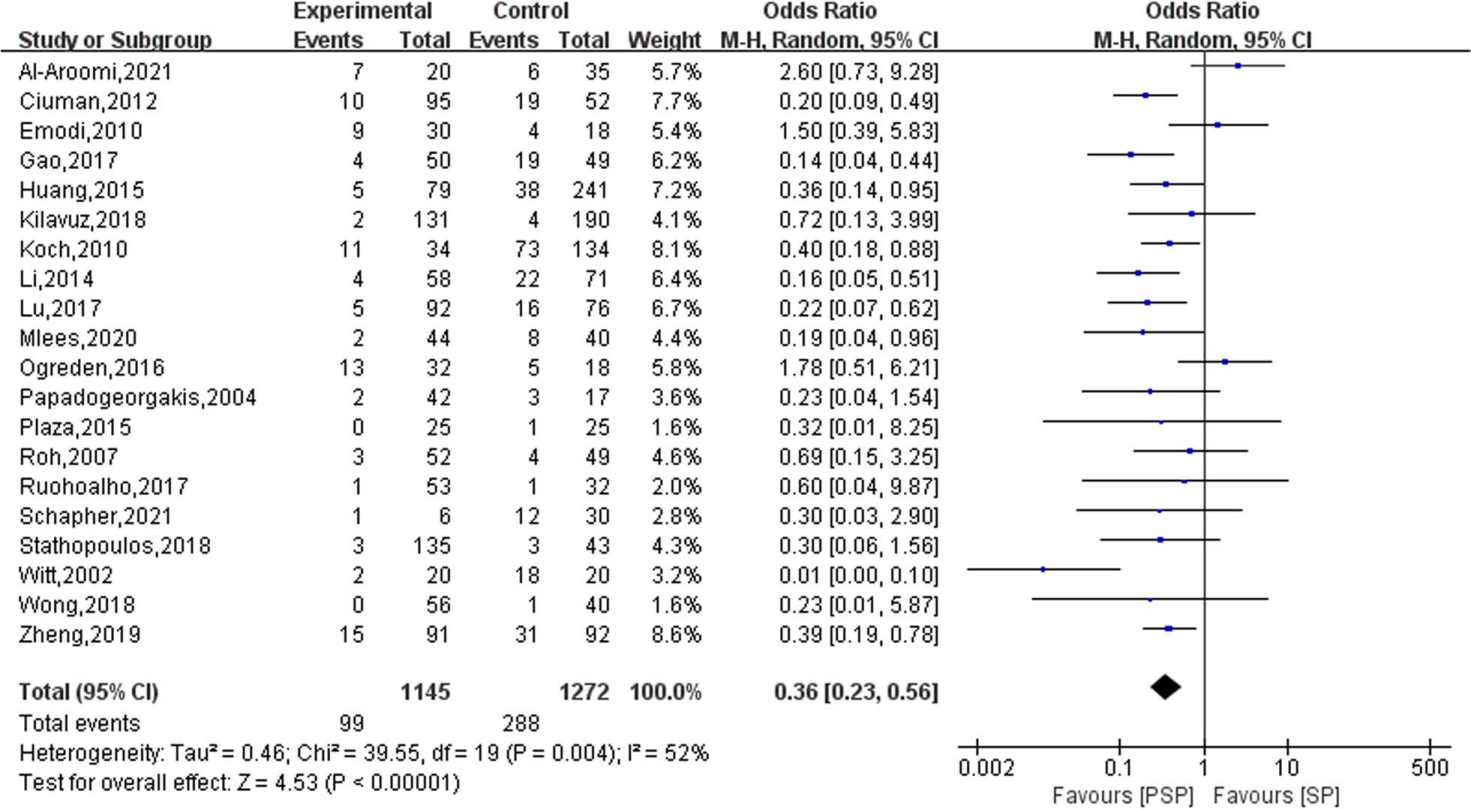
The effect of PSP and SP on Frey syndrome in patients

Twenty studies reported on the outcome of Frey syndrome occurring in the study subjects after surgery, with a total of 1,145 treated with PSP and a total of 1,272 treated with SP experiencing the syndrome. The results of the heterogeneity evaluation showed heterogeneity among the included studies (*I*^2^ = 52%, *P* = 0.004), and the combined effect size was calculated using a random effects model. The meta-analysis results showed that patients treated with PSP had a lower risk of developing Frey syndrome postoperatively compared to those treated with SP (OR = 0.36; 95% CI 0.23–0.56), as shown in Fig. 4. Sensitivity was analysed by presenting literature data one by one. When one study was excluded [29], the heterogeneity was reduced to 38%, and the combined effect size was 0.38 (95% CI 0.29–0.50) using a fixed effects model for the meta-analysis.

### Salivary fistula

**Fig. 5:**
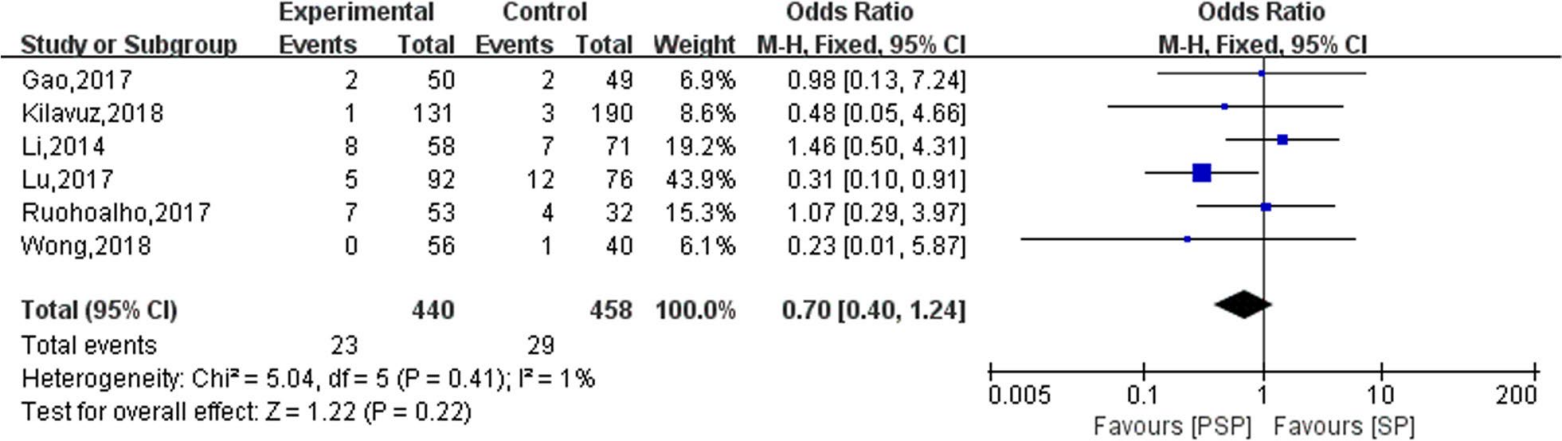
The effect of PSP and SP on patients with salivary fistula

Six studies reported on the outcome of salivary fistulas occurring in the study subjects after surgery. The results of the heterogeneity evaluation showed good homogeneity between the included studies (*I*^2^ = 1%, *P* = 0.41), and the meta-analysis was performed using a fixed effects model. The results of this study showed no statistically significant difference in PSP versus SP in the development of postoperative salivary fistula (OR = 0.70; 95% CI 0.40–1.24), as shown in Fig. 5.

### Recurrence rate

**Fig. 6:**
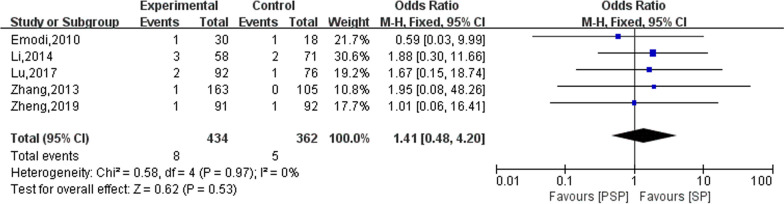
The effect of PSP and SP on the tumor recurrence rate of patients

Fifteen studies reported on the outcome of tumour recurrence in patients after surgery, of which 10 studies did not find tumour recurrence during follow-up [10, 20–24, 26, 28, 29, 34]. Therefore, only the remaining five studies were meta-analysed in this study. The results of the heterogeneity evaluation showed no heterogeneity among the included studies (*I*^2^ = 0%, *P* = 0.97), and the meta-analysis was performed using a fixed effects model. The final combined effect size showed that the effect of PSP and SP on postoperative tumour recurrence rate was not statistically significant (OR = 1.41; 95% CI 0.48–4.20), as shown in Fig. 6.

### Sialocele

**Fig. 7:**
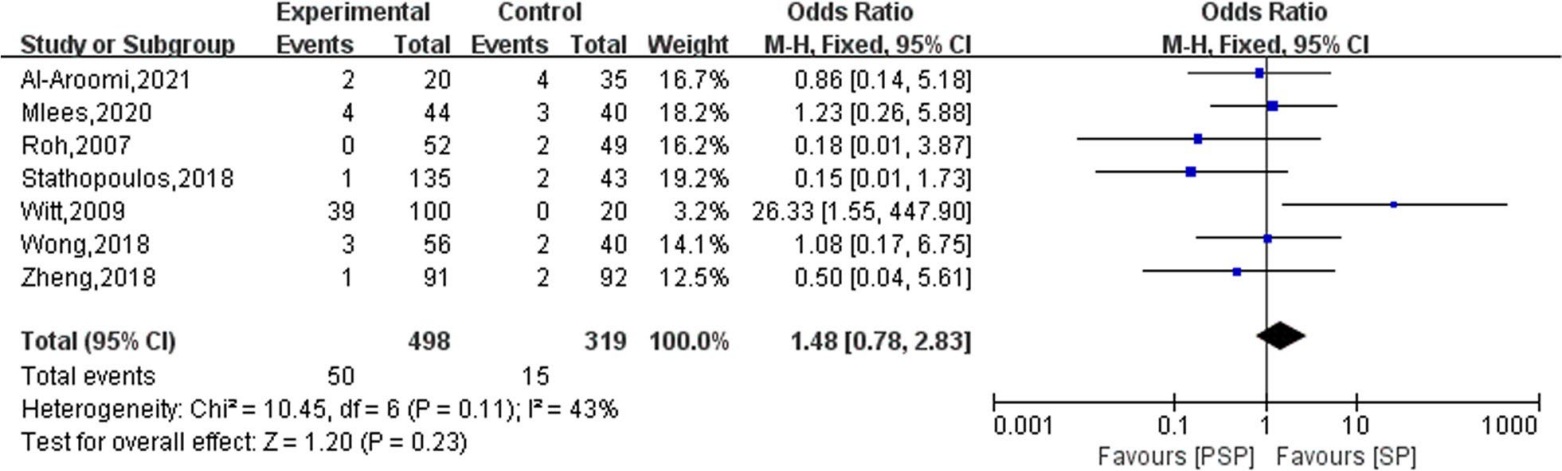
The effect of PSP and SP on sialocele in patients

Eight studies reported on the outcome of postoperative sialocele in the patients. Only one of them did not detect sialocele during follow-up [26], and the remaining seven studies were meta-analysed in this study. The results of the heterogeneity evaluation showed (*I*^2^ = 43%, *P* = 0.11) good homogeneity among the included studies, and the combined effect size was calculated using a fixed effects model. The results of the meta-analysis showed that the effect of PSP and SP on the occurrence of sialocele in patients after surgery did not show a statistically significant difference (OR = 1.48; 95% CI 0.78–2.83), as shown in Fig. 7.

### Haematoma

**Fig. 8:**
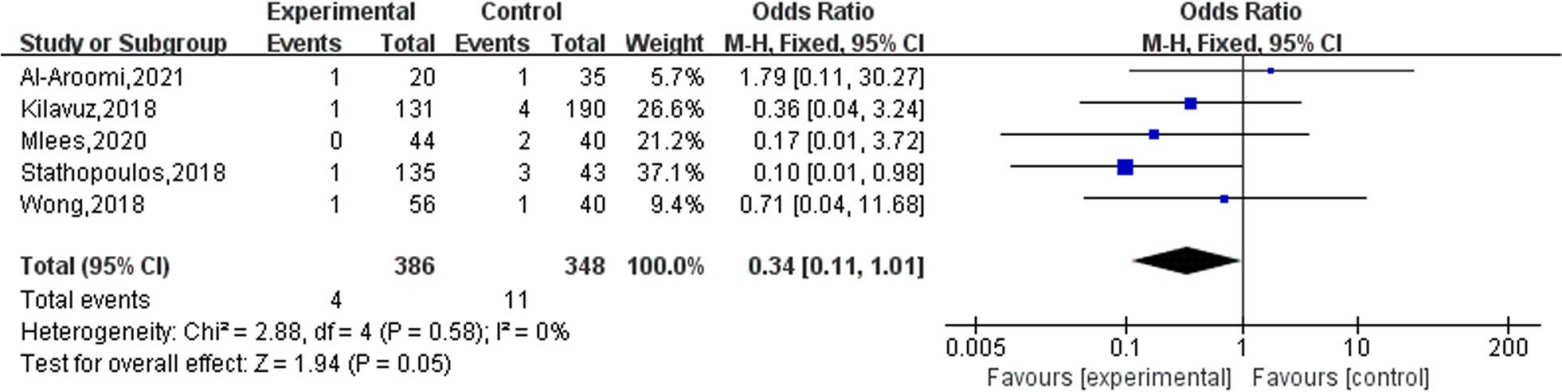
The effect of PSP and SP on the hematoma of patients

Five studies reported on the occurrence of postoperative haematomas in patients. The results of the heterogeneity evaluation showed no heterogeneity between the included studies (*I*^2^ = 0%, *P* = 0.58), and the meta-analysis was performed using a fixed effects model. The final combined effect size showed that the PSP treatment modality reduced the occurrence of postoperative haematoma in patients compared to SP (OR = 0.34), but this positive effect was not statistically significant (95% CI 0.11–1.01), as shown in Fig. 8.

### Surgery time

**Fig. 9:**
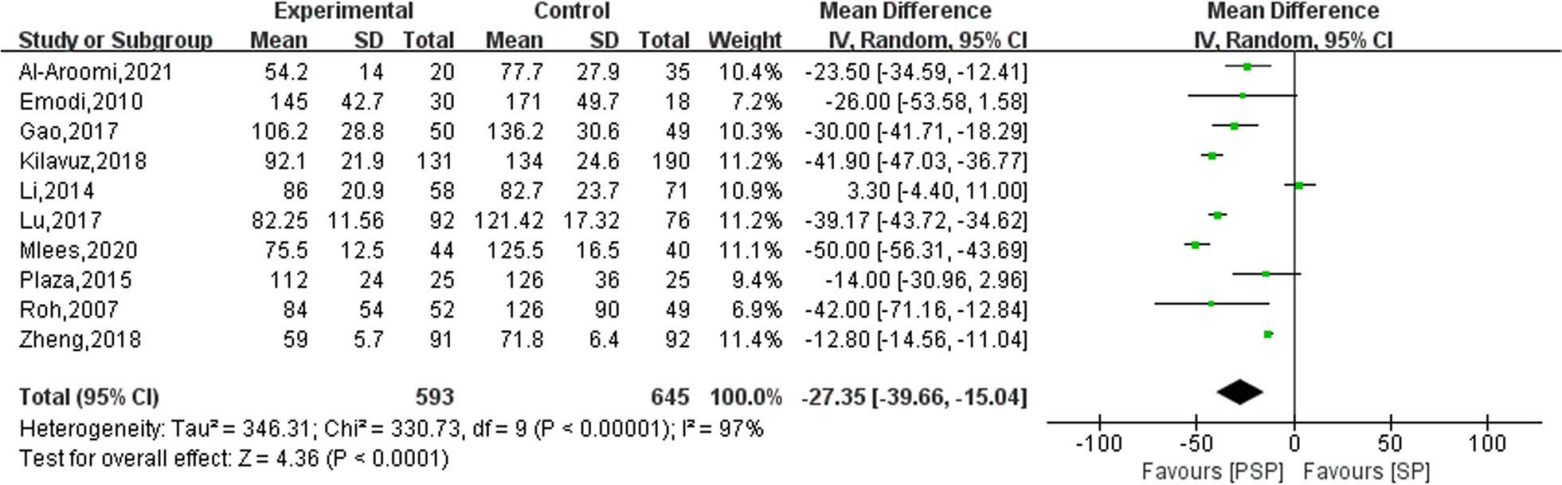
Comparison results of PSP and SP surgery time (min)

Ten studies reported on the operative time for PSP and SP. The results of the heterogeneity evaluation showed heterogeneity between the included studies (*I*^2^ = 97%, *P* < 0.00001), and the combined effect size was calculated using a random effects model. The results of the meta-analysis showed that the operative time of PSP was significantly lower than that of SP (MD: − 27.35; 95% CI − 39.66, − 15.04), as shown in Fig. 9. Upon excluding studies one by one for the sensitivity analysis, no significant sources of heterogeneity were found, indicating relatively stable heterogeneity between the included studies.

## Discussion

After a systematic search of English and Chinese databases, we systematically evaluated 23 high-quality studies comparing the PSP and SP surgical approaches. In this study, we found that the PSP surgical approach was beneficial to patients in reducing the incidence of facial nerve palsy (both temporary and permanent facial palsy) and Frey syndrome after surgery, and its operative time was significantly shorter. However, the effect of PSP versus SP on patients’ postoperative salivary fistula, sialocele, haematoma and tumour recurrence did not have any statistically significant difference. The results of this study provided evidence to compare the effects of PSP versus SP regarding the postoperative complications of benign parotid tumours and were consistent with the results of previous studies [13, 35].

The risk of postoperative complications in patients with benign parotid tumours may be related to the extent of surgical resection. The extent of parotidectomy and facial nerve stripping is usually determined by the size and location of the tumour and the preoperative diagnosis. An important consideration in parotid surgery is to preserve the facial nerve while achieving complete pathological resection and avoiding postoperative complications, such as Frey syndrome, facial palsy and salivary fistula [32, 36]. The extent of PSP is significantly less than that of SP, and Witt et al. [29] concluded that removing 1 cm of normal tissue outside the tumour reduces the recurrence rate to a great extent. Moreover, PSP does not affect the completeness of surgical resection, and the tumour recurrence rate is not significantly higher. The results of this study showed that the difference between the postoperative recurrence rates of PSP and SP was not statistically significant, indicating that PSP does not lead to an increase in recurrence rate by reducing the extent of resection of glandular tissue around the tumour. Additionally, PSP does not deliberately dissect the facial nerve, reducing the probability of facial nerve exposure and injury, so the risk of both transient and permanent facial palsy after PSP is lower than that after SP. For Frey syndrome, the main symptom of which is redness and sweating of the skin in the affected auriculotemporal region during eating, the incidence is 5–7% [37], and it occurs mostly within 5 weeks to 1 year after surgery [38]. Partial SP involves a small anatomical area and preserves the superficial musculoaponeurotic system on the surface of the gland as much as possible, forming a layer of mechanical safeguards to achieve the effect of preventing Frey syndrome.

In recent years, surgical techniques for benign parotid tumors have been developed in the anatomical direction of less invasive procedures [39]. Experienced salivary gland surgeons have taken this approach one step further by performing extracapsular dissection (ECD). An important aspect of ECD is that no dissection of the main trunk of the facial nerve is attempted. Previous showed that PSP had a higher rate of reported sensation abnormalities since the greater auricular nerve was divided, while the ECD had a significantly lower percentage of this postoperative complication [40]. However, due to the limitations that existed in previous studies, evidence exploring the effects of PSP and ECD were needed in the clinical practice.

The present study has some limitations. To start, the results of the heterogeneity evaluation regarding the duration of surgery were high, and the sensitivity analysis did not reveal a significant source of heterogeneity. This may have been due to the fact that the implementation of PSP treatment protocols requires patients to meet relevant indications and that surgeon experience and learning curves have varying degrees of influence on the implementation of PSP. In addition, the sample sizes of most studies were small, with only two studies involving more than 100 participants undergoing PSP or SP treatment. The smaller sample sizes may have resulted in statistically insignificant results for the studies of interest, leading to a lack of representativeness of the findings.

In conclusion, the results of this study provide evidence to compare the effects of PSP with SP on postoperative complications in benign parotid tumours. Partial SP treatment modalities outperform SP modalities in terms of the occurrence of temporary facial palsy, permanent facial palsy and Frey syndrome in the postoperative period, and the duration of surgery is shorter. However, because of the limitations of this study, a large number of high-quality studies are still needed in the future to investigate in depth the role of PSP in the postoperative effects on patients with benign parotid tumours.

## Data Availability

All data generated or analysed during this study are included in this article. Further enquiries can be directed to the corresponding author.

## References

[1] Zbären P, Stauffer E (2007). Pleomorphic adenoma of the parotid gland: histopathologic analysis of the capsular characteristics of 218 tumors. Head Neck.

[2] Tian Z, Li L, Wang L, Hu Y, Li J (2010). Salivary gland neoplasms in oral and maxillofacial regions: a 23-year retrospective study of 6982 cases in an eastern Chinese population. Int J Oral Maxillofac Surg.

[3] Bradley PJ, McGurk M (2013). Incidence of salivary gland neoplasms in a defined UK population. Br J Oral Maxillofac Surg.

[4] DeB Norman JE, McGurk M (1995). Color atlas and text of the salivary glands: diseases, disorders and surgery.

[5] Foresta E, Torroni A, Di Nardo F, de Waure C, Poscia A, Gasparini G, Marianetti TM, Pelo S (2014). Pleomorphic adenoma and benign parotid tumors: extracapsular dissection vs superficial parotidectomy—review of literature and meta-analysis. Oral Surg Oral Med Oral Pathol Oral Radiol.

[6] Albergotti WG, Nguyen SA, Zenk J, Gillespie MB (2012). Extracapsular dissection for benign parotid tumors: a meta-analysis. Laryngoscope.

[7] Iizuka K, Ishikawa K (1998). Surgical techniques for benign parotid tumors: segmental resection vs extracapsular lumpectomy. Acta Otolaryngol Suppl.

[8] Quer M, Guntinas-Lichius O, Marchal F, Vander Poorten V, Chevalier D, León X, Eisele D, Dulguerov P (2016). Classification of parotidectomies: a proposal of the European Salivary Gland Society. Eur Arch Otorhinolaryngol.

[9] Lu HB, Ma WN, Yu H, Sun L, Guo XL (2017). Retrospective study of partial superficial parotidectomy and superficial parotidectomy on superficial parotid benign tumor. Lin Chung Er Bi Yan Hou Tou Jing Wai Ke Za Zhi.

[10] Ogreden S, Ruzgar S, Alimoglu Y, Eroglu S, Taskin U, Oktay MF (2016). Comparison of Frey syndrome rates following superficial parotidectomy and partial superficial parotidectomy for pleomorphic adenoma. J Craniofac Surg.

[11] Al-Aroomi MA, Mashrah MA, Abotaleb BM, Sun J, Al-Worafi NA, Huang Y, Xie F (2021). Comparison of postoperative complications and facial nerve recovery rates after conventional and partial superficial parotidectomy of benign parotid tumours: a prospective study. Int J Oral Maxillofac Surg.

[12] Page MJ, McKenzie JE, Bossuyt PM, Boutron I, Hoffmann TC, Mulrow CD, Shamseer L, Tetzlaff JM, Akl EA, Brennan SE, Chou R, Glanville J, Grimshaw JM, Hróbjartsson A, Lalu MM, Li T, Loder EW, Mayo-Wilson E, McDonald S, McGuinness LA, Stewart LA, Thomas J, Tricco AC, Welch VA, Whiting P, Moher D (2021). The PRISMA 2020 statement: an updated guideline for reporting systematic reviews. BMJ.

[13] Zhang M, Jia ZY, Liu SY (2019). Evidence-based medical analysis of superficial partial lobectomy versus superficial lobectomy for benign parotid tumors. J Clin Otolaryngol Head Neck Surg.

[14] Stang A (2010). Critical evaluation of the Newcastle–Ottawa scale for the assessment of the quality of nonrandomized studies in meta-analyses. Eur J Epidemiol.

[15] Ruohoalho J, Mäkitie AA, Aro K, Atula T, Haapaniemi A, Keski-Säntti H, Takala A, Bäck LJ (2017). Complications after surgery for benign parotid gland neoplasms: a prospective cohort study. Head Neck.

[16] Zhang SS, Ma DQ, Guo CB, Huang MX, Peng X, Yu GY (2013). Conservation of salivary secretion and facial nerve function in partial superficial parotidectomy. Int J Oral Maxillofac Surg.

[17] Koch M, Zenk J, Iro H (2010). Long-term results of morbidity after parotid gland surgery in benign disease. Laryngoscope.

[18] Li C, Xu Y, Zhang C, Sun C, Chen Y, Zhao H, Li G, Fan J, Lei D (2014). Modified partial superficial parotidectomy versus conventional superficial parotidectomy improves treatment of pleomorphic adenoma of the parotid gland. Am J Surg.

[19] Ciuman RR, Oels W, Jaussi R, Dost P (2012). Outcome, general, and symptom-specific quality of life after various types of parotid resection. Laryngoscope.

[20] Stathopoulos P, Igoumenakis D, Smith WP (2018). Partial superficial, superficial, and total parotidectomy in the management of benign parotid gland tumors: a 10-year prospective study of 205 patients. J Oral Maxillofac Surg.

[21] Roh JL, Kim HS, Park CI (2007). Randomized clinical trial comparing partial parotidectomy versus superficial or total parotidectomy. Br J Surg.

[22] Mlees MA, Elbarbary AH (2020). Superficial or partial superficial parotidectomy for the treatment of primary benign parotid tumors. J Surg Oncol.

[23] Huang G, Yan G, Wei X, He X (2015). Superficial parotidectomy versus partial superficial parotidectomy in treating benign parotid tumors. Oncol Lett.

[24] Kilavuz AE, Songu M, Pinar E, Ozkul Y, Ozturkcan S, Aladag I (2018). Superficial parotidectomy versus partial superficial parotidectomy: a comparison of complication rates, operative time, and hospital stay. J Oral Maxillofac Surg.

[25] Emodi O, El-Naaj IA, Gordin A, Akrish S, Peled M (2010). Superficial parotidectomy versus retrograde partial superficial parotidectomy in treating benign salivary gland tumor (pleomorphic adenoma). J Oral Maxillofac Surg.

[26] Papadogeorgakis N, Skouteris CA, Mylonas AI, Angelopoulos AP (2004). Superficial parotidectomy: technical modifications based on tumour characteristics. J Craniomaxillofac Surg.

[27] Witt RL (2009). The incidence and management of siaolocele after parotidectomy. Otolaryngol Head Neck Surg.

[28] Plaza G, Amarillo E, Hernández-García E, Hernando M (2015). The role of partial parotidectomy for benign parotid tumors: a case-control study. Acta Otolaryngol.

[29] Witt RL (2002). The significance of the margin in parotid surgery for pleomorphic adenoma. Laryngoscope.

[30] Schapher M, Koch M, Goncalves M, Mantsopoulos K, Iro H (2021). Extracapsular dissection in pleomorphic adenomas of the parotid gland: results after 13 years of follow-up. Laryngoscope.

[31] Zheng CY, Cao R, Gao MH, Huang ZQ, Sheng MC, Hu YJ (2019). Comparison of surgical techniques for benign parotid tumours: a multicentre retrospective study. Int J Oral Maxillofac Surg.

[32] Wong WK, Shetty S (2018). The extent of surgery for benign parotid pathology and its influence on complications: a prospective cohort analysis. Am J Otolaryngol.

[33] Gao L, Ren W, Li S, Yan X, Li F, Yuan R, Shang W, Zhi K (2017). Comparing modified with conventional parotidectomy for benign parotid tumors. ORL J Otorhinolaryngol Relat Spec.

[34] Eski E, Sökmen MF, Yilmaz I (2018). Segmental superficial parotidectomy in the surgical treatment of benign parotid tumours. J Laryngol Otol.

[35] Li C, Matthies L, Hou X, Knipfer C, Gosau M, Friedrich RE (2020). A meta-analysis of the pros and cons of partial superficial parotidectomy versus superficial parotidectomy for the treatment of benign parotid neoplasms. J Craniomaxillofac Surg.

[36] Nouraei SA, Ismail Y, Ferguson MS, McLean NR, Milner RH, Thomson PJ, Welch AR (2008). Analysis of complications following surgical treatment of benign parotid disease. ANZ J Surg.

[37] Moltrecht M, Michel O (2004). The woman behind Frey's syndrome: the tragic life of Lucja Frey. Laryngoscope.

[38] Bonanno PC, Palaia D, Rosenberg M, Casson P (2000). Prophylaxis against Frey's syndrome in parotid surgery. Ann Plast Surg.

[39] Psychogios G, Bohr C, Constantinidis J, Canis M, Vander Poorten V, Plzak J, Knopf A, Betz C, Guntinas-Lichius O, Zenk J (2021). Review of surgical techniques and guide for decision making in the treatment of benign parotid tumors. Eur Arch Otorhinolaryngol.

[40] Laskaris S, Chrysikos D, Koutrafouris I, Piagkou M, Protogerou V, Karampelias V, Bekos F, Kotzias D, Troupis T (2022). Partial superficial parotidectomy versus extracapsular anatomical dissection for the treatment of benign parotid tumors. Acta Med Acad.

